# Psychological aspects in young people with venous thromboembolic disease, preliminary report

**DOI:** 10.1016/j.htct.2024.06.007

**Published:** 2024-09-08

**Authors:** Alejandro Godoy, N. Bula Galli, Aldo Tabares

**Affiliations:** a Vascular Medicine and Thrombosis Service, Hospital Privado Universitario Córdoba, Argentina; b Psychiatric Service, Hospital Privado Universitario Córdoba, Argentina; c Instituto de Ciencias Biomédicas de Córdoba (IUCBC), Argentina

**Keywords:** Venous thromboembolism, Mental health, Anxiety, Depression, Post-traumatic stress

## Abstract

**Background:**

The decline in the mental well-being of young adults following an episode of venous thromboembolism may be related to the uncertainty of long-term health and fear of recurrence. In recent years, post-pulmonary embolism syndrome has gained acceptance, however, less attention has been given to the psychological impact on young patients after venous thromboembolism. This study explores the prevalence, type, and severity of psychological disorders of patients following venous thromboembolism.

**Methods:**

A retrospective observational cohort study was performed of over 18-year-old patients diagnosed with venous thromboembolism followed in the Vascular Medicine Service at Hospital Privado de Córdoba, Argentina from July 2020 to October 2021. Due to the COVID-19 pandemic, virtual interviews were conducted using two pre-established questionnaires administered by the same psychiatrist. The first questionnaire gathered personal data, clinical history, and mental health information, while the second, evaluated mood disorders using the Mini International Neuropsychiatric Interview. Patients with a positive MINI score underwent further assessment using the Hamilton Scale. Patients were considered young if ≤45 years.

**Results:**

A total of 50 patients were assessed, 56 % were women, and 54 % were ≤45 years. Major depression was documented in 11 (22 %) patients, eight (72 %) in the younger group, and three (28 %) in the older group. Eight (16 %) patients had an anxiety disorder, four in the younger group, and ten (20 %) patients had post-traumatic stress disorder, seven (70 %) of the younger patients. Generalized anxiety disorder was identified in 20 (40 %) patients with similar proportions in both groups.

**Conclusion:**

Psychological and emotional symptoms are common following an episode of venous thromboembolism. Post-traumatic stress disorder and depression appear to be numerically more prevalent in the young.

## Introduction

Pulmonary embolism (PE), affecting an estimated 600,000 to 1 million individuals annually, ranks as the third leading cause of cardiovascular mortality worldwide, following myocardial infarction and stroke.^1^^,^^2^

PE carries a significant one-year mortality rate of 15 %, with survivors frequently enduring persistent physical symptoms such as exercise limitations, breathlessness, and post-thrombotic syndrome. Although there have been considerable advancements in the management of venous thromboembolism (VTE), addressing the psychological impact in patients remains a crucial clinical challenge.^3^^,^^4^

Emotional well-being profoundly influences health outcomes in conditions like coronary artery disease, diabetes, hypertension, and chronic obstructive pulmonary disease.^5^ Similarly, a VTE diagnosis is often associated with emotional distress, mental fatigue, anxiety, and depression, with many patients fearing recurrence. Despite of this, the full extent of the psychological impact of VTE is not yet fully understood.^6^

Research consistently shows that PE patients experience higher rates of anxiety and depression compared to the general population.^7^ In young adults with VTE, reduced mental well-being often correlates with concerns about their future health and the possibility of recurrence, resulting in a notably high usage of psychotropic medications. Within five years after the diagnosis of VTE, approximately 20 % of teenagers and young adults have been prescribed psychotropic medications, a rate double that of their age and gender-matched peers.^8^^,^^9^

Persistent dyspnea and functional limitations significantly contribute to ongoing anxiety among PE survivors. Age is a critical factor, as younger patients often experience heightened anxiety related to the loss of independence and increased dependence on healthcare systems. In contrast, older patients may better manage these challenges due to their accumulated life experiences and developed coping mechanisms. ^10^

Qualitative research involving PE survivors highlights significant psychological effects, including post-traumatic stress symptoms such as intrusive thoughts, flashbacks, and hypervigilance. While several studies have utilized validated measures to assess anxiety and depression in this population, they have mostly been cross-sectional with limited sample sizes. Nonetheless, these studies reveal elevated depression and anxiety scores compared to controls, and significant psychological distress not present before the diagnosis. ^11^

Common challenges among VTE patients include managing symptoms, feelings of vulnerability, altered self-perception, and heightened vigilance. Despite these findings, the trajectory of mental health following PE remains poorly understood, and there is a lack of evidence-based strategies to improve mental health outcomes for these patients. ^12^

This study aims to address this critical gap by examining the prevalence of psychological disorders in both young and adult patients receiving care at a tertiary medical institution.

## Methods

An observational cohort study was initiated involving over 18-year-old individuals who presented for consultations for VTE at Hospital Privado Universitario de Córdoba and were subsequently assessed by the Vascular Medicine Service.

**Figure 1:**
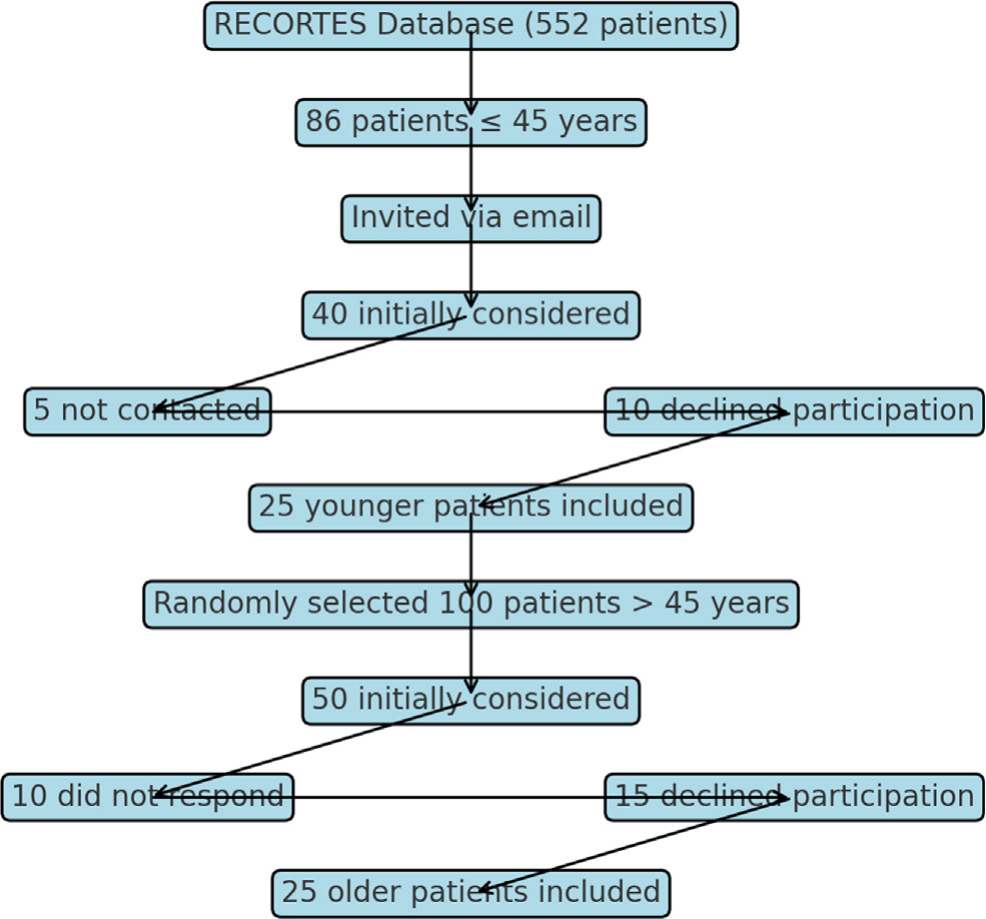
Flowchart of patient selection.

Under 45-year-old patients were selected from three years (2017–2020) from the RECORTES (Retrospective Córdoba Thromboembolism Study) database, which registered VTE cases diagnosed at the hospital from January 2008 to December 2020. Invitations to participate were sent via email, and those who accepted were included in the study. The RECORTES registry includes 552 patients, of which 86 are younger than 45 years old. From 2017 to 2020 a total of 40 under 45-year-old patients were initially considered, however five could not be contacted and ten declined to participate, leaving 25 participants. During the same period, 100 over 45-year-old patients (controls) were considered, and two control patients were randomly assigned for each younger patient, stratified by the year of the VTE episode. Of the 50 selected older patients, ten did not respond, and 15 declined to participate, leaving a total of 25 participants (Figure 1).

A series of 50 virtual interviews were conducted from July 2020 to October 2021 with two pre-established questionnaires administered by a designated psychiatrist from the hospital's mental health service. The first questionnaire gathered personal data, clinical history, and mental health information and the second evaluated mood disorders using the Mini International Neuropsychiatric Interview (MINI).^13^ Patients with a positive MINI score underwent further assessments with the Hamilton Anxiety Rating Scale (HAM-A) and the Hamilton Depression Rating Scale (HDRS). ^14^^,^^15^

The MINI aimed at diagnosing a range of mood disorders. It is known for its brevity and diagnostic precision, identifying 16 core psychiatric disorders in line with the Axis I classification of the DSM V. The MINI poses no risk to the interviewer and does not interfere with or alter the psychological or social environment of the patient. It assesses depressive disorders, dysthymia, suicide risk, hypomanic episodes, panic disorder, agoraphobia, social anxiety disorder, obsessive-compulsive disorder, post-traumatic stress, substance abuse and dependence, psychotic disorders, anorexia nervosa, bulimia nervosa, generalized anxiety disorder, and antisocial disorder. Typically, a healthcare professional can administer the questionnaire within a 15-minute window.

The Hamilton psychic and somatic scale was utilized for individuals with a positive score in the MINI. This externally administered 14-item tool primarily focuses on anxiety indicators, with 13 items dedicated to gauging anxious manifestations and one item assessing the individual's demeanor during the interview. Each item is scored from 0 to 4 by the interviewer, reflecting both its intensity and occurrence. Cumulative scores range from 0 to 56 points. Distinct scores can be derived for psychic anxiety (items 1–6 and 14) and somatic anxiety (items 7–13). Levels of generalized anxiety are delineated as 0–5 (No anxiety), 6–14 (Minor anxiety), 15+ (Major anxiety); 14+ is Clinically evident anxiety.

The HDRS was employed in cases where depression was diagnosed. This externally administered tool specifically tailored for those with a prior depression diagnosis, offers a quantitative measurement of symptom intensity and tracks variations. The compact 17-item variant endorsed by the US National Institute of Mental Health and validated in Spanish by Ramos-Brieva was chosen for this study.^16^

Multiple assessments vouch for its discriminant validity, reliability, and sensitivity across both inpatient and outpatient settings. Each item offers 3–5 potential responses, scored between 0 and 2 or 0–4. Aggregate scores span from 0 to 52. Depression severity is demarcated using thresholds advised by the Clinical Practice Guide of the National Institute for Health and Care Excellence (NICE): 0–7 (not depressed), 8–13 (Mild depression), 14–18 (Moderate depression), 19–22 (Severe depression), and 23+ (Very severe depression).

Study participants were stratified into two distinct categories: those under 45 years diagnosed with either DVT or PE and those 45 years and older.

For data analysis, categorical variables are reported using frequencies and percentages, while continuous variables are expressed as mean values and standard deviations. The Chi-square test was employed for categorical distinctions and the T-test or Wilcoxon test was used for continuous variables. The analysis also encompassed the development of multiple logistic regression models.

## Results

A total of 50 virtual meetings were conducted, 56 % were women, and 54 % of the patients were under 45 years old. The average age in the cohort was 45.4 years. Twenty-two patients (44 %) were evaluated within six months after the initial event. The patient cohort was followed from January 2017 through 2022. The average length of follow-up was two years after the event.

Regarding the marital status of the population, 40 % were single, 40 % were married, 11 % were divorced, and 9 % were widowed.

Concerning the educational level, 76 % had complete secondary education, 22 % had incomplete secondary education, and 2 % had primary education.

For ten patients (20 %) the thrombotic event was unprovoked, while for the remainder, it could be biologically and/or temporally correlated with thrombosis-causing factors (Table 1).

**Table 1:** Patient distribution by venous thromboembolism condition.

Condition	N
Unprovoked	10
Congenital vascular malformation	1
Clinical hospitalization	3
Polytrauma	2
Oral contraceptives	7
Cardiac tumor	1
Oncological disease	8
Postpartum	1
Pregnancy	1
Cardiac catheterization	1
Antiphospholipid syndrome	1
Prolonged bed rest	3
Post-abdominal surgery	6
Thrombophilia	1
COVID	5
Hepatorenal transplant	1
Renal transplant	1

In all cases, the proposed treatment was oral anticoagulation. Some patients were still undergoing treatment at the time of the interview, while others had completed the prescribed duration and were continuing with outpatient follow-ups; 40 (80 %) patients had experienced provoked events.

Eleven patients (22 %) suffered from major depression, eight of whom were under the age of 45 compared with three patients in the older age group (p-value = 0.189; relative risk [RR]: 0.44; 95 % confidence interval [95 % CI]: 0.13–1.4). Eight patients (16 %) presented with distress disorder (currently panic disorder), four young patients and four older patients (RR: 1.17; 95 % CI: 0.33–4.17). Twenty percent had post-traumatic stress disorder, seven belonged to the young group and three to the adult group (p-value: 0.3; RR: 0.5; 95 % CI: 0.14–1.7). Twenty patients (40 %) had generalized anxiety, half of whom were young (p-value = 0.77; RR: 1.17; 95 % CI: 0.59–2.31).

No significant associations were found between the presence of mood disorders and the variables analyzed in the multivariate analysis.

**Figure 2:**
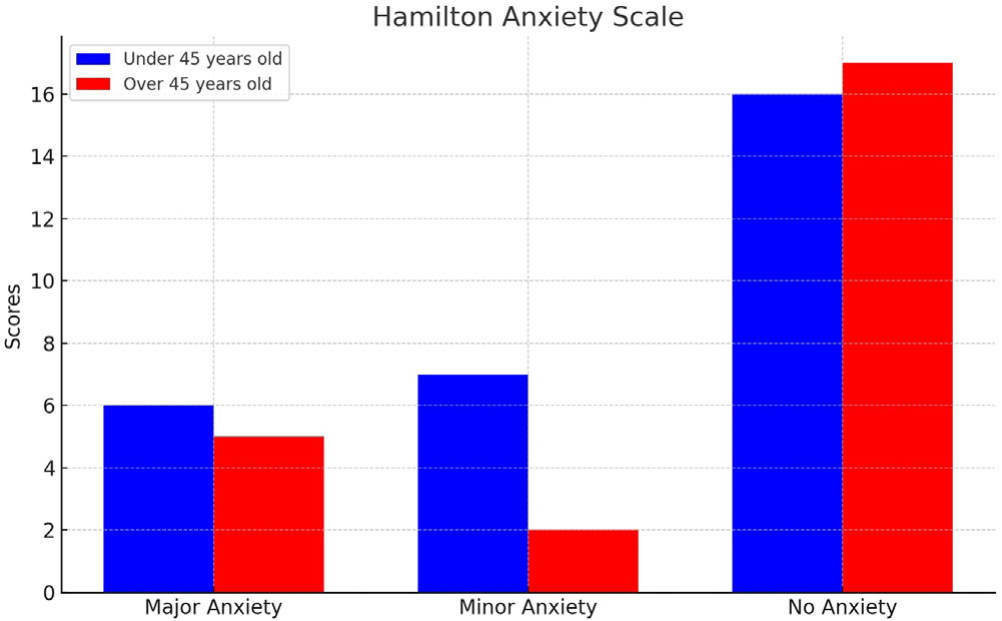
Hamilton anxiety scale.

For under 45-year-old patients, anxiety (according to the HAM-A) was minor in 26 % and major in 22 %, compared with 13 % and 17 % in the over 45-year-old cohort. This difference was not statistically significant as indicated by the chi-square test (p-value = 0.900 - Figure 2).

**Figure 3:**
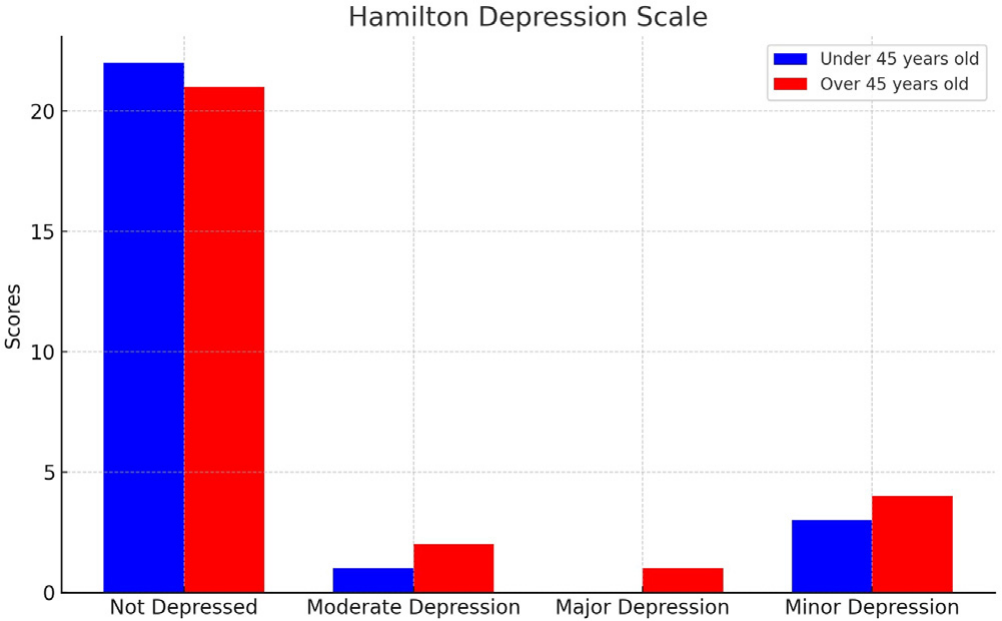
Hamilton depression scale.

Based on the HDRS, 15 % and 7 % presented with minor and moderate depression, respectively in under 45-year-old patients compared with 4 % and 7 % in older patients. No major depression diagnoses were observed, and there were no statistically significant differences between the variables analyzed (Figure 3).

Of the 50 patients, 29 reported receiving psychological or psychiatric assessments and treatment. Notably, four of these patients initiated their mental health treatment after experiencing VTE events and were all younger than 45 years old. In addition, there were five patients aged over 45 who were found to be taking psychotropic medication without formal indications or supervision by a mental health professional. Most of the diagnoses reported during the interview were mood disorders (Depression, Anxiety, and Bipolar Disorder), and in one case, the reason for follow-up was to control a psychotic episode secondary to the use of high doses of corticosteroids in the context of an admission for renal transplantation. Additionally, 12 patients had previously consulted with a psychologist for reasons other than the thrombotic event, and seven had both treatments, four of whom had had both before suffering DVT and/or PE, and in one of these cases, their symptoms recurred after the thrombotic event; three patients began psychological treatment for the first time after the event.

A numerical but non-significant trend was observed in emergency medical consultations for symptoms compatible with VTE in under 45-year-old patients (75 % versus 25 %; p-value = 0.08; RR: 0.39; 95 % CI: 0.12–1.27).

As for other complications stemming from this condition that were sought through outpatient consultations, the most frequent (eight responses) was pain and edema of limbs. Other complications mentioned included bleeding due to anticoagulant treatment, limited physical activity, arrhythmia, weakness, and late recurrences of the disease.

## Discussion

Mood symptoms following an acute VTE episode are common. Although we could not show a significant statistical difference in mood disorders between under and over 45-year-old patients, there is a trend toward these disorders being more frequent in individuals under 45. A higher rate of emergency consultations was also observed in younger patients.

This study reveals a significant prevalence of psychological disorders among young adults who have experienced VTE. Major depression was documented in 22 % of patients, with a notably higher prevalence in the younger cohort (72 % of the depressed patients were under 45 years old). Additionally, 16 % of patients were found to have anxiety disorders, and 20 % experienced PTSD. Generalized anxiety disorder was present in 40 % of patients, with similar proportions in both younger and older age groups. These findings highlight the profound mental health burden that accompanies VTE, particularly in younger individuals.

A recent Danish study explored the association between VTE and the subsequent risk of depression. Utilizing nationwide registries, researchers formed a cohort comprising 64,596 individuals with incident VTE, which they compared to a cohort of 322,999 individuals from the general population. The findings demonstrated a markedly increased incidence of depression within the VTE cohort, with 44.4 cases per 1000 person-years, compared to 19.4 cases per 1000 person-years in the general population cohort. The absolute risk of depression was found to be 10.3 % in the VTE cohort versus 5.6 % in the general population, translating to 4.7 excess cases of depression per 100 individuals with VTE. VTE was associated with a 2.35-fold increased risk of depression relative to the general population, a risk that persisted even after adjustments for socioeconomic status and comorbidities, yielding a hazard ratio of 1.91. The study further noted that PE and cancer-provoked VTE were linked to a higher risk of depression, with PE showing a 2.31-fold increased risk and cancer-provoked VTE demonstrating a 2.96-fold increased risk when compared to the risk in the general population. The temporal pattern of the association between depression and VTE was evident, with the strongest association observed during the initial years following a VTE diagnosis.^17^

In the first three months following a PE diagnosis, patients experience a significantly reduced quality of life, though a notable improvement is observed during the follow-up. Despite this gradual improvement, their long-term quality of life continues to be lower than that of the general population. This persistent decline is influenced by factors such as fear of recurrence, advanced age, cancer, and cardiovascular comorbidities.^6^

In a prospective, single-center case-control study, researchers assessed the impact of VTE on mental health in 163 patients with confirmed VTE and 55 control subjects using the Zung Self-Rating Anxiety Scale and Zung Self-Rating Depression Scale. The study showed that patients with VTE had notably higher anxiety and depression scores compared to the control group; the mean scores for anxiety were 33.98 ± 9.93 in the VTE group versus 28.53 ± 6.96 in the control group, and for depression, they were 38.22 ± 11.22 versus 32.42 ± 7.6, respectively, with both differences being statistically significant. Additionally, the study highlighted gender and age-related effects, showing that women and younger patients faced a higher risk for developing these mental health issues after VTE. A significant correlation was also found between anxiety and depression across all participants, indicating that these conditions are commonly comorbid regardless of VTE occurrence. ^18^

A study involving 50 young patients who had experienced their first thrombotic episode 3–18 months before compared them with 39 healthy controls matched for age, sex, and educational background using seven questionnaires assessing self-esteem, family and social functioning, and coping strategies. Results indicate that young thrombosis patients exhibit significantly lower self-esteem and have poorer social interactions and family relationships than healthy controls. They also rely more on coping strategies, particularly passive ones like avoidance and religiosity. This trend is especially pronounced among women and those under 34, who are more psychologically affected.^10^

Multiple factors contribute to the increased levels of depression and anxiety observed in VTE patients. Firstly, PE acts as a significant stressor that can trigger various pathophysiological and psychological responses. Anxiety and depression often emerge shortly after the diagnosis of acute physical conditions. The dyspnea and sense of impending suffocation frequently experienced by VTE patients due to arterial hypoxemia significantly contribute to these mood disturbances. Studies have shown a direct relationship between dyspnea and the development of anxiety and depression symptoms.^19^ VTE, being an acute and life-threatening event, causes significant functional impairment, early retirement from work, and a reduced quality of life. Long-term anticoagulant therapy can adversely affect lifestyle due to the heightened risk of bleeding, potentially leading to depression either as a psychological response to the diagnosis or as a side effect of the treatment. Qualitative studies indicate that functional deterioration—such as pain, swelling, reduced mobility, and complications like recurrence, post-thrombotic syndrome, and post-pulmonary embolism syndrome—also contributes to depression. Experiencing a major PE is often described as a life-changing event, with patients reporting a loss of identity and life roles. The fear of imminent death during the diagnosis often results in persistent post-traumatic fear of recurrence. ^20^

Patients report feeling permanently altered by their experience, with physical and psychological reminders of the PE. Trauma and anxiety symptoms are prevalent, with many patients reporting that daily life is negatively influenced and expressing intense concern about not recognizing symptoms of a recurrent PE in time. There is also significant fear that their symptoms might be underestimated or not taken seriously by healthcare providers.

Although the present study is constrained by a relatively small sample size and potential self-selection bias, the findings underscore the need for further research with larger, more diverse cohorts. Future studies should also focus on the long-term psychological effects of VTE and develop tailored interventions for younger patients.

Despite these limitations, this study sheds light on the psychological burden of VTE, particularly in young adults. Many interviews were conducted during the COVID-19 pandemic, which may have exacerbated existing anxieties. Additionally, the time elapsed between the VTE event and the interview varied, though psychological distress appeared persistent.

Given the high prevalence of psychological disorders among young VTE patients, it is essential to implement comprehensive care plans that include mental health support. Regular psychological assessments and early interventions, such as cognitive behavioral therapy, counseling, and psychoeducation, should be integrated into standard follow-up protocols to optimize patient well-being and treatment adherence.

Healthcare providers must implement a holistic approach that addresses both the physical and psychological sequelae of VTE, ensuring timely interventions and continuous support.

Future research should expand to include large-scale, longitudinal studies to elucidate the long-term psychological consequences of VTE and rigorously evaluate the efficacy of various interventions. These investigations should specifically focus on the unique needs of young VTE patients, developing tailored mental health strategies that address their specific vulnerabilities. Furthermore, understanding how VTE and its treatment contribute to psychological disorders will help develop more effective prevention and management strategies.

## Conclusion

This study demonstrates the significant psychological impact of VTE on young adults, highlighting the need for integrated mental health care in managing VTE. Addressing the psychological consequences of VTE is crucial for enhancing patients' quality of life and reducing the risk of recurrence. The findings of this study emphasize the importance of considering mental health in the overall treatment and follow-up of VTE patients, particularly younger individuals who may experience more profound psychological effects.

## Conflicts of interest

The authors declare no conflicts of interest.
